# Quantum dot-sensitized solar cells having 3D-TiO_2_ flower-like structures on the surface of titania nanorods with CuS counter electrode

**DOI:** 10.1186/s11671-015-0844-0

**Published:** 2015-03-21

**Authors:** Nattha Buatong, I-Ming Tang, Weeraphat Pon-On

**Affiliations:** Department of Physics, Faculty of Science, Kasetsart University, 50 Ngam Wong Wan Road, Lat Yao Chatuchak, Bangkok, 10900 Thailand; Department of Material Science, Faculty of Science, Kasetsart University, 50 Ngam Wong Wan Road, Lat Yao Chatuchak, Bangkok, 10900 Thailand

**Keywords:** Quantum dot-sensitized solar cell (QDSSC), Nanostructures, Semiconductors, Counter electrode

## Abstract

The photovoltaic performance of a quantum dot (QD)-sensitized solar cell consisting of CdS/CdSe/ZnS QDs loaded onto the surface of the three-dimensional (3D) flower-like TiO_2_ structure grown on an array (1D) of TiO_2_ nanorods (FTiR) is studied. The flower-like structure on the rod-shaped titania was synthesized using a double-step hydrothermal process. The FTiR array exhibited a 3D/1D composite structure with a specific surface area of 81.87 m^2^/g. Using CuS as the counter electrode instead of Pt offers the best performance and leads to an increase in the conversion efficiency (*η*). The efficiency of the CdS/CdSe/ZnS QD-loaded FTiR assembling CuS counter electrode cell improved from *η* = 2.715% (*V*oc = 0.692 V, *J*sc = 5.896 mA/cm^2^, FF = 0.665) to *η* = 0.703% (*V*oc = 0.665 V, *J*sc = 2.108 mA/cm^2^, FF = 0.501) for the QD-loaded FTiR assembling Pt counter electrode cell. These studies reveal a synergistically beneficial effect on the solar-to-current conversion of these QD-sensitized solar cells when a CuS counter electrode is used instead of the usual Pt counter electrode.

## Background

Demand for energy has been increasing. Since most of this energy is produced by burning fossil fuel, the resulting environmental pollution has rapidly increased. This has resulted in the air that people breathe becoming detrimental to their health. Also, the amount of clean fossil fuel has been decreasing. An alternative source of energy is the sun. As long as man is alive, this will be an unlimited source. In recent years, there have been several reports [1-3] on how solar energy can be developed so that it can become the alternative source of energy. For this to occur, new ways to convert sunlight to useable energies must be found.

Photovoltaic conversion of solar-to-electrical energy is one of these methods. One of the more promising conversion technologies is the use of dye-sensitized solar cells (DSSCs). These consist of organic dye molecules adsorbed on a wide band gap of semiconductor such as TiO_2_, and much research has gone into fabricating and developing these DSSCs [4-6].

Recently, the use of quantum dots to extend the absorption spectrum of TiO_2_ has been proposed. This has lead to the development of quantum dot-sensitized solar cells (QDSSCs) [7-15]. They are considered to be a new generation of photovoltaic devices. The QDs exhibit unique optical and electrical properties based on them having tunable band gap across a wide range of energy levels. Earlier reports [10,11] have focused on the QDs such as CdS, CdSe, PbS, and PbSe or any combinations of them or on the electrolytes in the cells.

Another factor that should be considered is the morphology of TiO_2_-based photoanode materials. There have been several studies on how the morphology affects the conversional efficiency of solar cell [12-15]. A mesoporous structure TiO_2_ nanoparticle film has a higher surface area. This increases the amount of semiconducting quantum dots that can be loaded [12]. However, the increase in the amount of surface defects and grain boundaries in the film would greatly retard the electron transport needed for the electron recombination process. To solve this problem, arrays of one-dimensional (1D) nanostructure (rod, wire tube) can be formed. It is generally believed however that a 1D structure (through a combination of a reduced recombination (achieving high electron-transfer rate) process and lower surface area) would have reduced solar-to-electrical conversion efficiencies [13].

Recently, composites consisting of mesoporous (or 3D hierarchical) structures of TiO_2_ grown on the individual TiO_2_ rods forming into a layered array have been reported [16-20]. The morphology of these composite allows for the high specific areas needed for greater sensitizer adsorption, remarkable light scattering ability, and void space for electrolyte infiltration which in turn would improve the solar energy harvesting (and the conversion efficiency). The replacement of the dye with the QDs to extend the spectrum range of solar absorption requires that different electrolytes be used in the cell. A typical electrolyte for QDSSCs would be the redox polysulfide couple (S*n*^2−^/S^2−^) (DSSCs is I_3_^−^/I^−^). Commercial Pt counter electrode would not be suitable for these QDSSCs since the strong chemiadsorption of the sulfide ions in three of the QDs mentioned would result in low connectivity between the two electrodes and this in turn would lead to decreasing catalytic activity [12]. In this regard, the studies on the metal sulfide, such as CuS, CoS, Cu_2_S, and composite electrodes (CuS/CoS) as counter electrodes, have been reported [21-26]. All of the studies reported that there were increases in the electrocatalytic activity and noticeable improvement in the power conversion efficiency when sulfide-based counter electrode was used in place of a Pt counter electrode.

**Figure 1:**
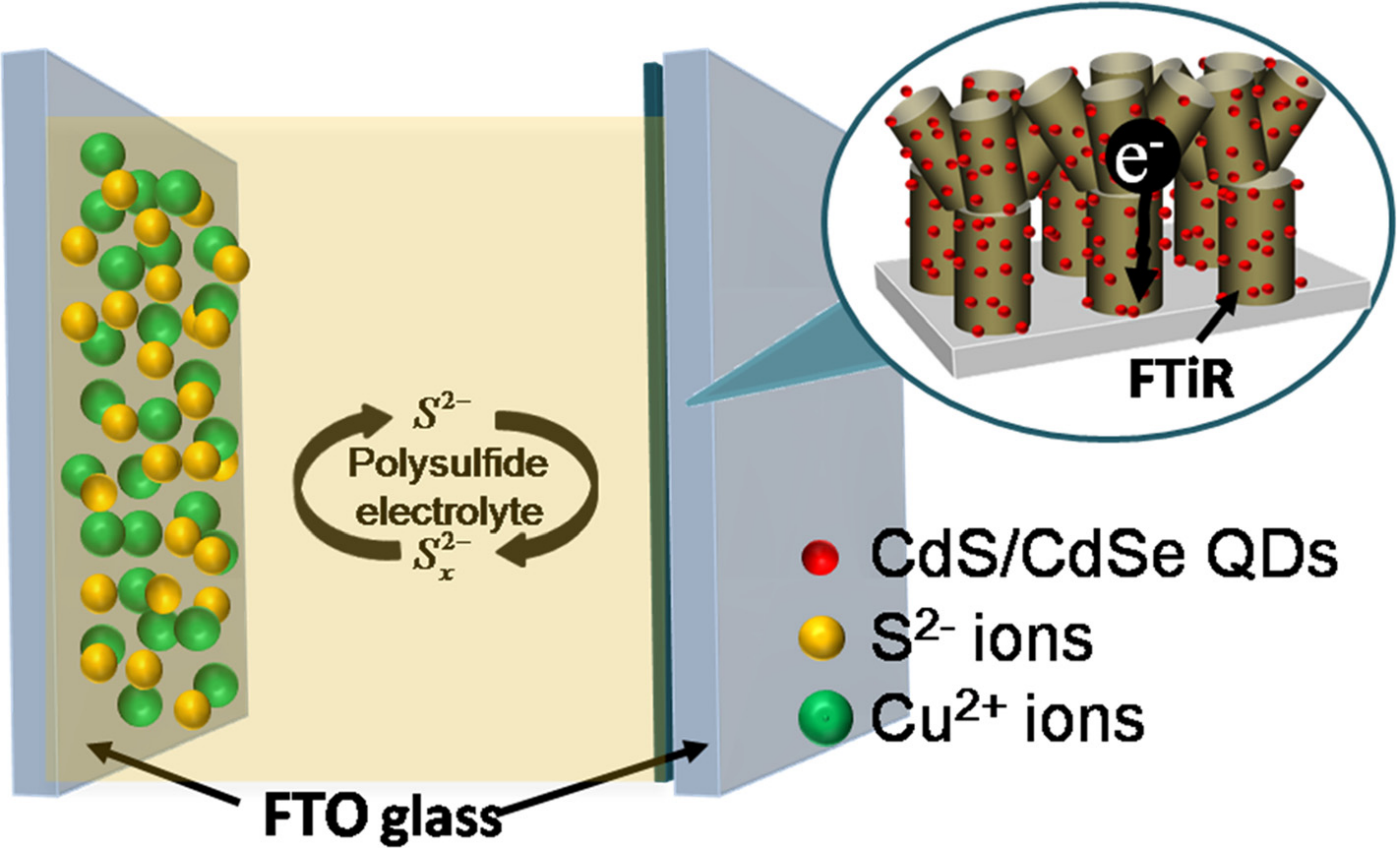
**A schematic diagram of the photovoltaic performance of a quantum dot (QD)-sensitized solar cell.** A schematic diagram of the photovoltaic performance of a quantum dot (QD)-sensitized solar cell consisting of CdS/CdSe/ZnS QDs loaded onto the surface of the three-dimensional (3D) flower-like TiO2 structure grown on an array (1D) of TiO2 nanorods (FTiR) assembling CuS counter electrode.

In the present study, we have studied the loading of semiconducting QDs onto the surface of three-dimensional flower-like titania structures grown on one-dimensional array of TiO_2_ nanorods formed on a conducting fluorine-doped tin oxide (FTO) glass substrate. Flower-like structures on the rod-shaped titania layer (FTiR) were synthesized via two-step hydrothermal technique. This formation of 3D flower-like structures on the 1D titania rods of FTiR (acting as the photoanode) would lead to a greatly increased surface area needed for the QD loading. This would lead to a greater light harvesting, resulting in a greater solar-to-electric conversion. CuS was used as a counter electrode in order to improve the conversion efficiency of the QDSSCs. The use of CuS instead of Pt to create the FTiR photoanode needed to bring about the redox reaction of polysulfide studied is shown in Figure 1. The structure, morphologies, and optical properties of the photoanodes were characterized by X-ray diffraction (XRD), scanning electron microscopy (SEM), transmission electron microscopy (TEM), and UV–vis spectroscopy. The QD-sensitized FTiR solar cells were assembled with CuS or Pt as the counter electrodes. The effect of using CuS or Pt in the QDSSCs on the photovoltaic performance on the open circuit voltage (*V*oc), short circuit current density (*J*sc), fill factor (FF), and efficiency (*η*) were investigated. By combining the CdS/CdSe/ZnS QD-loaded FTiR assembling CuS counter electrode cell leads to an *η* of 2.715%.

## Methods

### Preparation of FTiR on FTO substrates

In the present work, TiO_2_ films with arrays of nanorods having flower blossom-like structure surfaces formed on fluorine-doped tin oxide (FTO) glass substrates were successfully obtained via two-step hydrothermal technique. The structure was synthesized using a modification of method given in a previous report [27]. TiO_2_ rod arrays were firstly synthesized followed by a flower blossom-like structure in the second step. Before the synthesis process started, the FTO glass substrates were ultrasonically cleaned in a mixture of deionized water, acetone, and 2-propanol bath for 30 min and then blown dry with a flux of air immediately. To obtain the TiO_2_ rod arrays on the FTO glass substrates, a mixture of 12 mL of deionized water and 12 mL of HCl solution was stirred for 5 min. Then, 0.4 mL of titanium butoxide was added and stirred at room temperature until the solution became clear. The precursor solution at this point contained the colloidal TiO_2_ nanoparticles. A previously cleaned FTO glass substrate is placed in the precursor solution. This was then placed in a Teflon-lined stainless steel autoclave. The TiO_2_ nanoparticles were converted into nanorods by a hydrothermal reaction carried out at the temperature of 150°C for 20 h inside the oven. After the oven was cooled to room temperature, the substrates were taken out, rinsed with deionized water and ethanol, and dried in a desiccator.

Synthesis of an FTiR substrate which consisted of arrays of titania nanorods whose surface is covered by flower blossom-like formations was done in a second-step hydrothermal reaction. The TiO_2_ arrays on FTO glass (obtained from one-step hydrothermal process) were placed in the Teflon-lined stainless steel autoclave filled with a mixture containing 10 mL of toluene, 0.4 mL of titanium butoxide, and 1 mL of HCl and heated at 150°C for 5 h. After the process was completed, the oven was cooled to room temperature. Then, the samples were taken out, rinsed by deionized water and ethanol, and dried in a desiccator. The as-prepared substrates were heat treated at 450°C for 1 h before loading the quantum dots.

### Preparation of CdS/CdSe/ZnS quantum dot-sensitized FTiR substrates

Decoration of CdS/CdSe/ZnS quantum dots on FTiR substrates having an effective area of 0.25 cm^2^ was achieved by successive ion layer absorption and reaction (SILAR) and chemical bath deposition (CBD) using a modification of the procedure described in a previous report [28]. The CdS was deposited on the substrates by SILAR method by immersing the FTiR substrates into a solution containing Cd(NO_3_)_2_ · 4H_2_O (0.5 M) for 1 min, rinsing in deionized water and drying on a hot plate. The dried substrates were then dipped into a solution of 0.5 M Na_2_S · 9H_2_O aqueous solution for 5 min, rinsed with methanol, and dried on a hot plate. The process was repeated five times. These as-prepared electrodes will be referred to as the CdS photoanodes. The deposition of CdSe particles onto CdS photoanode structure was done using the CBD technique in which the CdS/FTiR were dipped into an aqueous solution of Cd(NO_3_)_2_ · 4H_2_O(0.5 M):Na_2_SeSO_3_(0.08 M):NH_4_OH (45 mM) having a solution temperature of 95°C for 3 h followed by rinsing with ethanol and drying on a hot plate. These as-prepared electrodes will be denoted as the CdS/CdSe photoanodes. To reduce the charge recombination between the quantum dots and the electrolyte, a ZnS layer was coated onto the CdS/CdSe/FTiR surfaces by immersing the last surfaces into a Zn(NO_3_)_2_ · 6H_2_O solution (0.1 M) for 1 min, rinsed with ethanol, and dried on a hot plate. They were then dipped for 5 min into 0.5 M Na_2_S aqueous solution, followed by rinsing with methanol and drying on a hot plate. The process was repeated three times. After the CdS/CdSe/ZnS decoration process is completed, the QD-sensitized arrays of titania nanorods whose surface was covered by flower blossom-like formations blossom-like formation were post-annealed at 450°C for 1 h under ambient air condition.

### Preparation of CuS counter electrodes

CuS counter electrode was prepared using a doctor-blade method according to the literature [29] with a fluorine-doped tin oxide (FTO) glass as substrates. Briefly, 100 μL of 0.5 M Cu(NO_3_)_2_ in methanol solution was dropped onto the FTO glass. The doctor-blade method was used to remove the excess Cu(NO_3_)_2_ on the FTO. Then, 100 μL of 1 M Na_2_S · 9H_2_O water–methanol (1:1 volume ratio) aqueous solution was dropped uniformly onto the Cu(NO_3_)_2_-decorated FTO. Upon dropping, the color changed from blue (of Cu(II)) to brown, implying the formation of CuS. The remainder of the ions was removed by rinsing with deionized water and drying using air gun under atmosphere. The two-step dropping, rinsing, and drying procedures were repeated two times. The film was calcinated at 450°C for 30 min under ambient air condition and finally cooled down to room temperature.

### Fabrication of quantum dot-sensitized solar cell

The QDSSCs were assembled into sandwich-like cell by using one of the CdS/CdSe/ZnS as the photoelectrode and one of the CuS/FTO as the counter electrode (for comparison, a second cell was made with Pt/FTO as the counter electrode was also assembled). The two electrodes were then placed in a thermoplastic biphenyl frame (Surlyn, DuPont, Wilmington, DE, USA). This sandwich was then annealed at 100°C for 25 min. The electrolyte in these solar cells was a polysulfide solution: 2.0 M Na_2_S, 0.5 M Na_2_SO_4_, and 0.2 M KCl in methanol/water (7:3, v/v). Two holes were made in the counter electrode, and a drop of the polysulfide electrolyte was put onto the hole. The polysulfide was introduced into the cell via vacuum backfilling. Finally, the hole was sealed using a Ti foil.

The properties of the QDSSCs employing the FTiR photoanodes were measured as follows. The photocurrent densities (*J*) and photo voltages (*V*) of the cells having an active area of 0.25 cm^2^ on the FTiR photoanodes were measured under AM 1.5 G simulated sunlight produced by a 150 W Class A Solar Simulator (Model 92250A, Oriel) at an illumination intensity of 100 mW/cm^2^. The incident light intensity was calibrated with a standard crystalline silicon solar cell (Oriel reference cell, 91,550 V). A power source meter (Keithley 2400) was used to measure the response of the solar cells.

### Characterizations

The crystalline phase of the samples was characterized by X-ray diffraction (XRD, Bruker D8 Advance, Bruker, Billerica, MA, USA). Diffraction patterns were recorded in the range of 20° to 80° with a scanning step of 0.02° s^−1^. The morphology and structure of the samples were investigated by scanning electron microscopy (SEM, JEOL JSM-6301 F, JEOL, Akishima-shi, Tokyo, Japan) and transmission electron microscopy (TEM, JEOL JSM-2010). The samples for TEM measurements were scraped from the substrate and dissolved in ethanol, followed by transferring one drop onto a carbon-coated copper grid. The specific surface areas of FTiR substrate was determined by the nitrogen adsorption-desorption isotherm measurement (Autosorb-cl analyzer, Quantachrome Instruments, Boynton Beach, FL, USA). The total pore volume was determined at (*P*/*P*_0_) 0.99. The adsorption spectra were recorded by UV–vis spectrophotometer (Perkin Elmer Lambda 900, Perkin Elmer, Waltham, MA, USA). The current–voltage (*I-V*) characteristics of solar cells were obtained by a potentiostat, and the cells were irradiated under AM 1.5 G illumination with an intensity of 100 mW cm^−2^.

## Results and discussion

### Characterizations of the photoelectrodes

**Figure 2:**
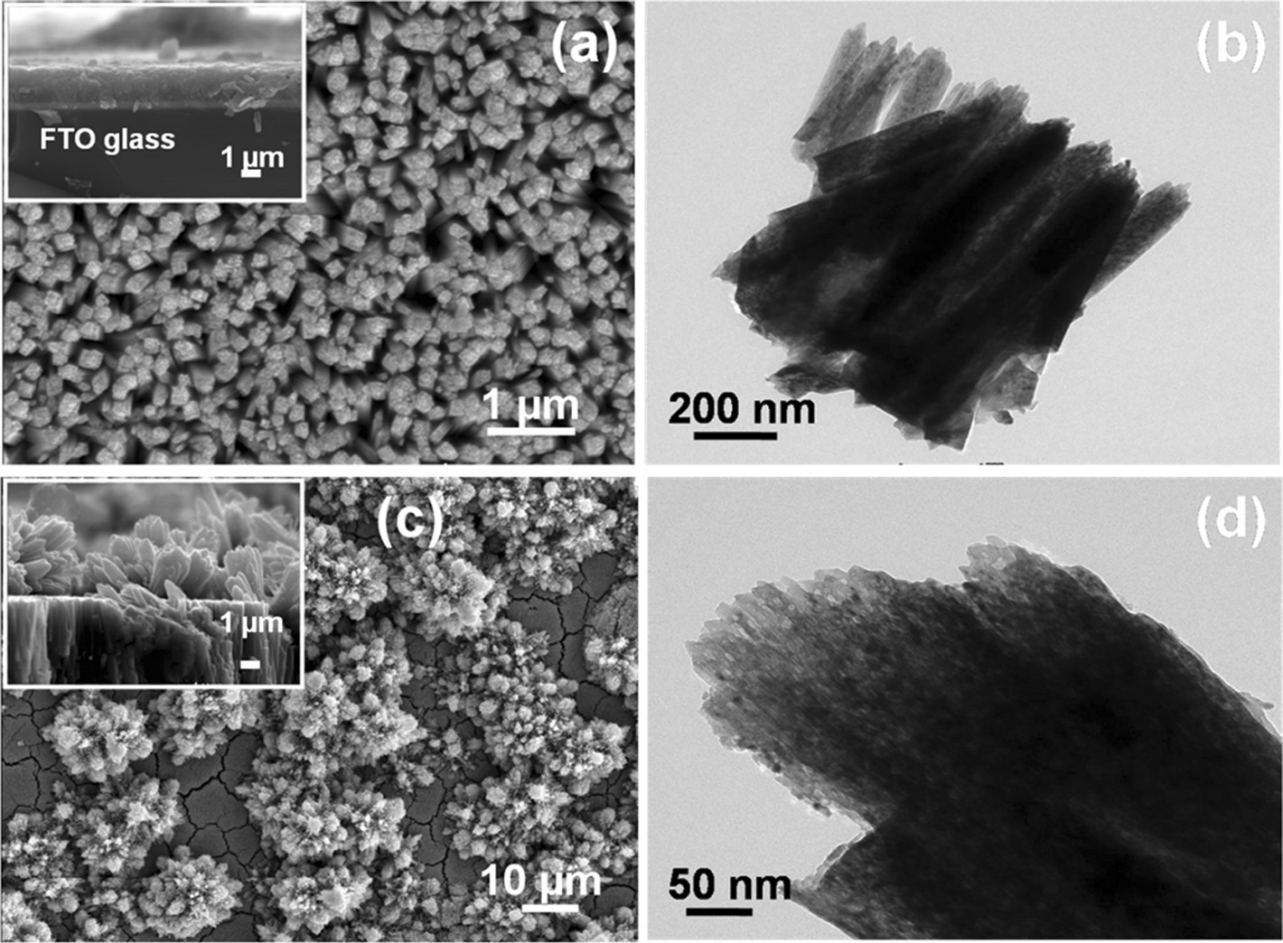
**SEM and TEM images of TiO**_**2**_ **nanorods and blossom-like formations on TiR surface. (a,** **b)** SEM and TEM images of TiO_2_ nanorods (TiR). **(c,** **d)** SEM and TEM images of (flower) blossom-like formations on TiR surface (FTiR).

The successful synthesis of TiR and FTiR on a FTO glass substrate can be seen in the SEM and TEM micrograms shown in Figure 2. Figure 2a shows the SEM image of the top of the TiR film, while the insert shows the cross-sectional view. Figure 2b is the TEM image of the TiR themselves. The typical lengths and diameters of the individual nanorods were between 3 and 5 μm and between 100 and 200 nm, respectively. After the second hydrothermal treatment, the appearance of flower blossom-like formation is seen. Figure 2c shows the top view while, the insert shows the cross-sectional view. Both the top and cross-sectional views show the blossom-like structured growth on each nanorod. Figure 2d is the TEM image of the nanorods in the FTiR array. The TEM images show that the nanorods in the TiR material (Figure 1b) have formed into a single layer of thickness of a few micrometer and that the nanorods in the FTiR material (Figure 2d) form into a double layer of aligned (array) rod-like objects. The latter image shows the lengths of the newly formed rod-like particles with a size range from 10 to 50 μm with the blossom-like formations on top of the rods of the original layer (see the insert in Figure 2c). The newly grown particles have a nanoplate-like structure.

**Figure 3:**
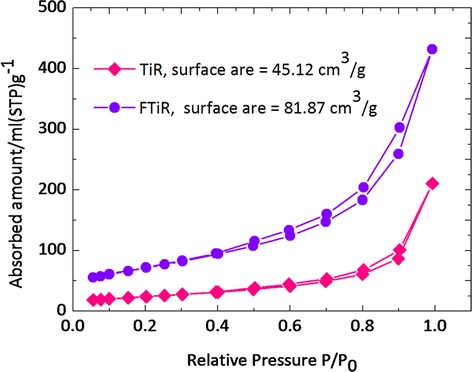
**The nitrogen adsorption-desorption isotherms of TiR and FTiR substrates.**

The results on the surface areas of the TiR and FTiR substrates were determined by the nitrogen adsorption-desorption isotherm measurements. The results of these measurements are shown in Figure 3. The amount of nitrogen gas absorbed at different partial pressure can be translated into the surface area. The results for the FTiR substrates gave a BET (Brunauer-Emmettt-Teller) surface area of 81.87 m^2^/g while the results of the TiR substrate gave a surface area of 45.12 m^2^/g. The increase in the surface area of the FTiR means that the formation of three-dimensional blossom flower-like formation of TiO_2_ on top of the one-dimensional titania nanorods would increase the surface area of the photoelectrode. This allows for a greater adsorption of the quantum dots.

**Figure 4:**
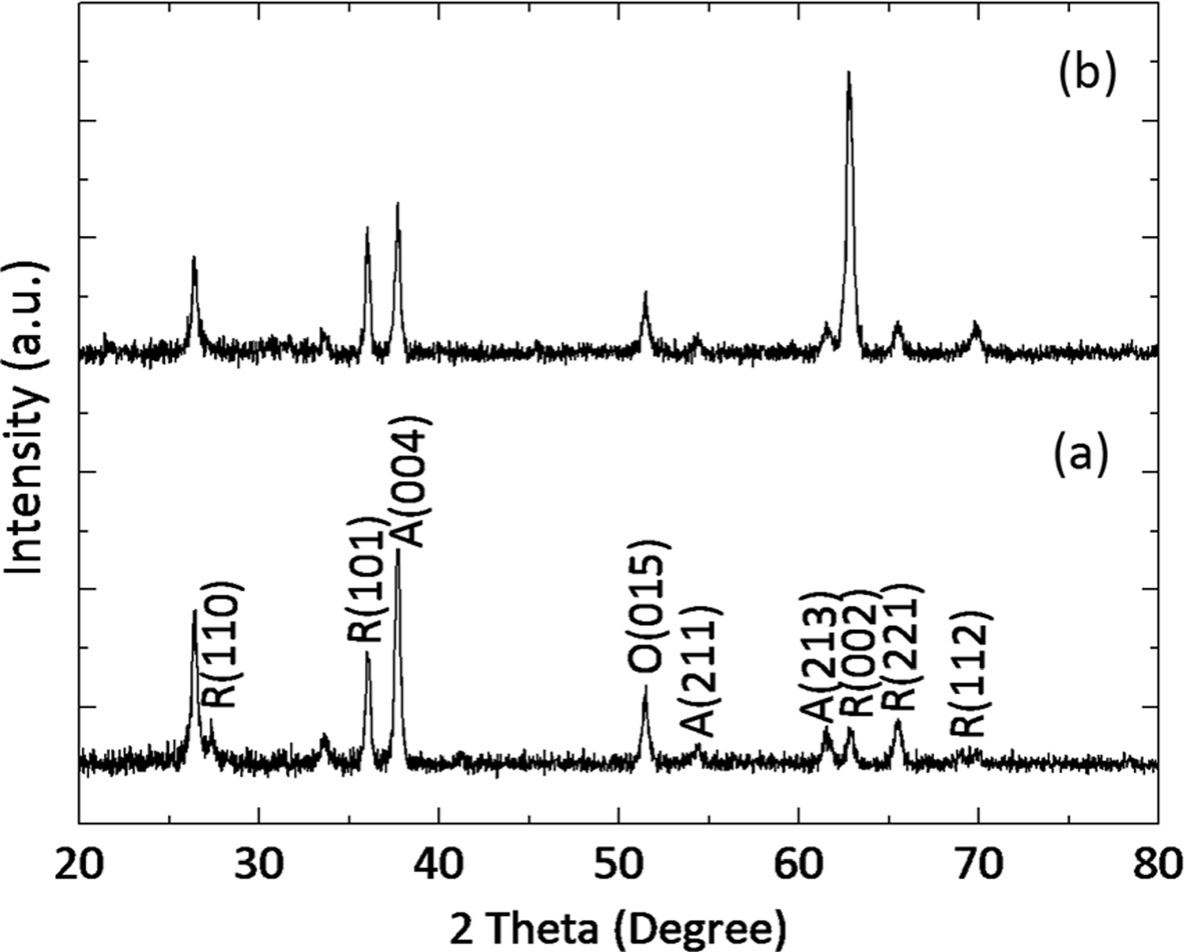
**The XRD pattern of (a) TiR and (b) FTiR substrates.**

The crystallization of FTiR substrates can be seen in Figure 4. The XRD patterns indicate that the main phase is the rutile phase (JCPDS 01-075-2545), and the minor phase is the anatase phase (JCPDS 01-075-1749). The diffraction intensity was significantly higher in the rutile phase on the FTiR substrate with the highest intensity peak being at 2*θ* ~ 62.5° (002). This is evidence that the c-axis is the preferred direction of growth.

**Figure 5:**
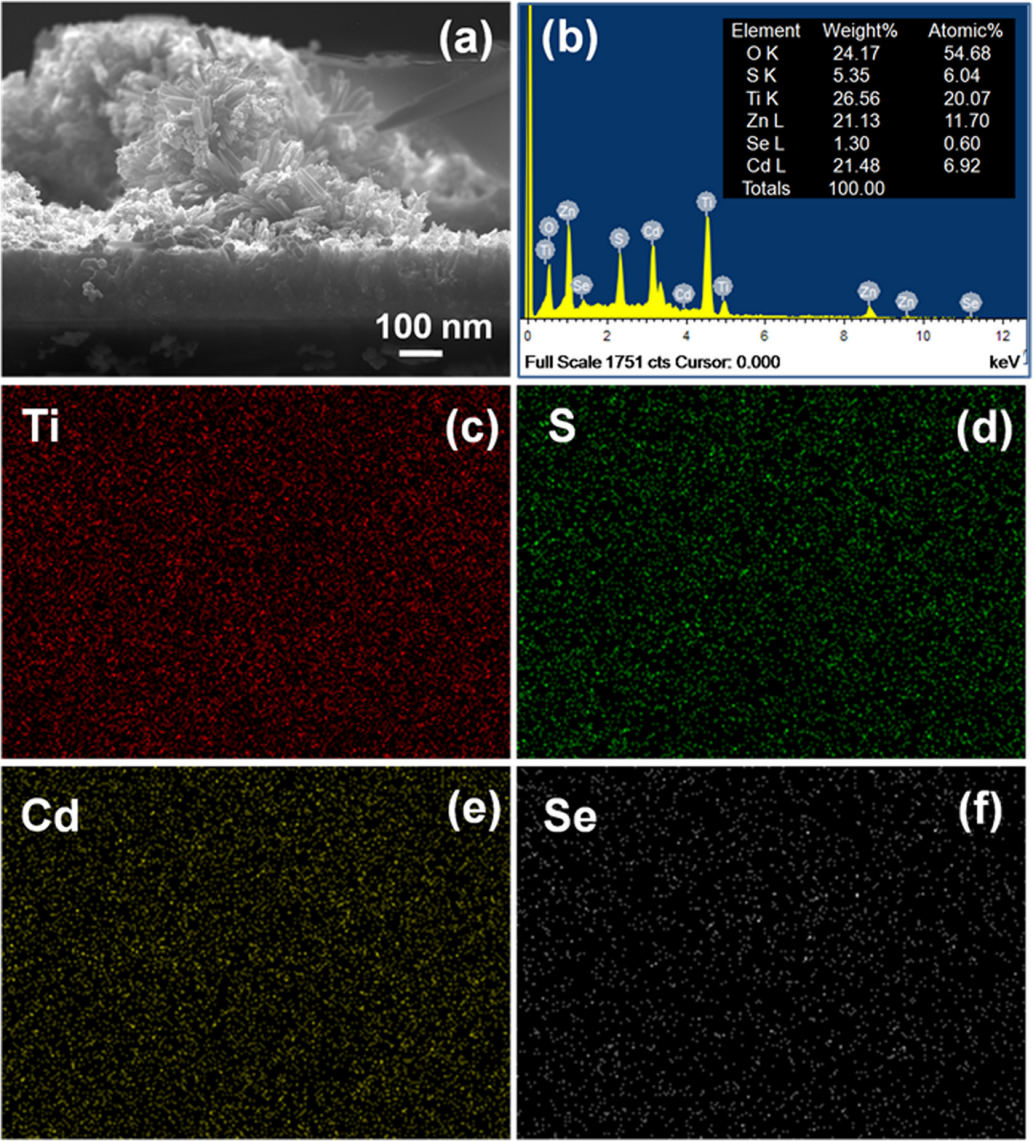
**SEM images of CdS/CdSe/ZnS QDs, EDS analysis of ion species, and element distribution maps. (a)** SEM images of CdS/CdSe/ZnS QDs on FTiR substrates and **(b)** EDS analysis of the deposited ion species of the QDs on FTiR substrates. Element distribution maps of **(c)** Ti, **(d)** S, **(e)** Cd, and **(f)** Se.

The deposition of CdS/CdSe/ZnS QDs on FTiR substrates was carried out by SILAR and CBD method. After QDs decorated the FTiR structure, a large number of CdS/CdSe/ZnS QDs filled the interspaces in the FTiR structure (as shown in Figure 5a). The composition of the deposited ion species of the QDs was determined by the EDS of the field emission scanning electron microscope (FE-SEM) (Figure 5b). Ti, O, Cd, S, Se, and Zn were detected, and their concentrations are 26.56%, 24.17%, 21.48%, 5.35%, 1.30%, and 21.13% by weight. These results confirmed that the QDs had been deposited on the FTiR substrates. Recordings (Figure 5c, f) of the EDS emissions at different wavelengths showed that the different elements were uniformly deposited on the substrates.

**Figure 6:**
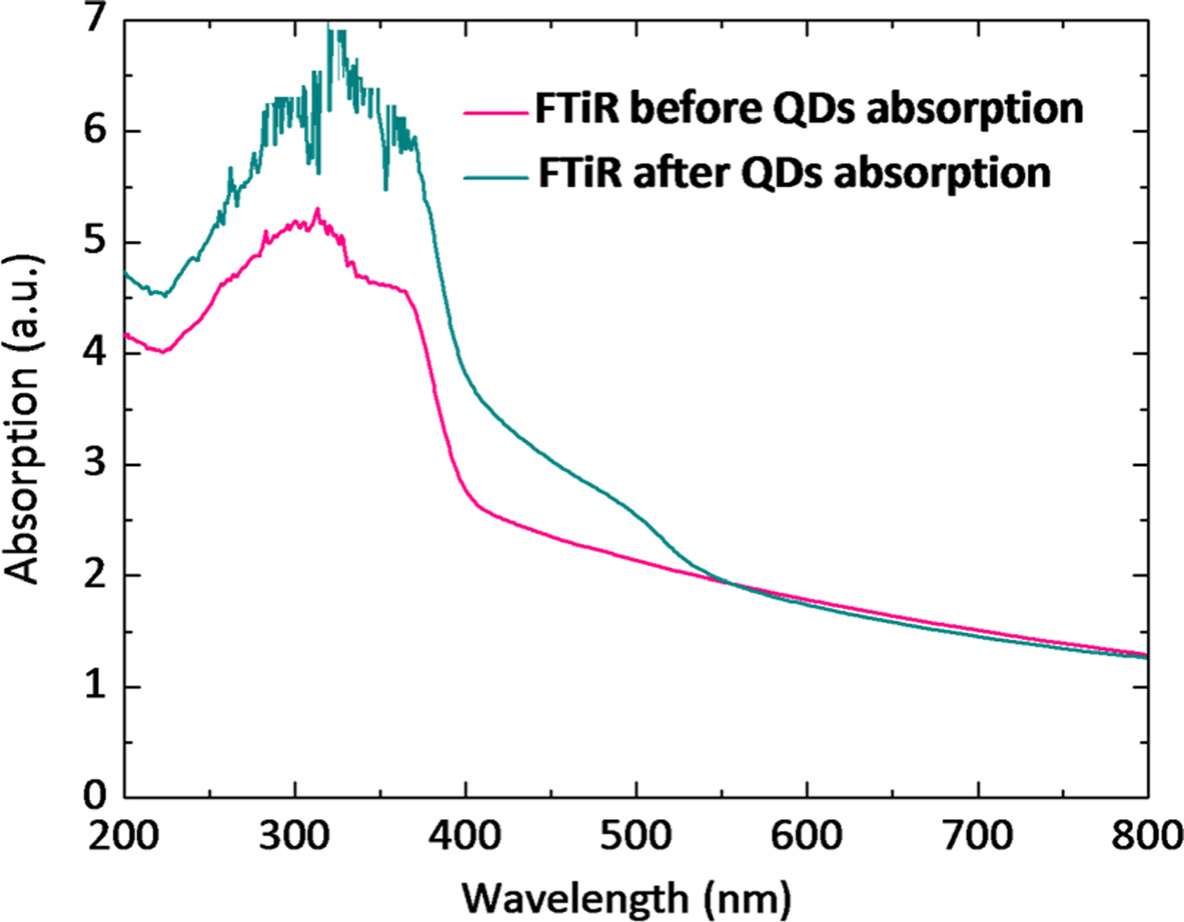
**UV–vis absorptive spectra of the photoanodes.**

The UV–vis absorption spectra of the CdS/CdSe/ZnS QD-sensitized photoelectrodes made with FTiR substrates are seen in Figure 6. It is obvious that the light absorption is shifted to longer wavelengths (red shift). The spectrum indicates that incident photons of lower energies can be utilized by the QDSSCs with the CdS/CdSe/ZnS QDs. The UV–vis spectra exhibits a cut-off edge of the nanorod single-layer film spectrum at around 350 nm, which corresponds to a band gap of 3.54 eV, which is much larger than that of bulk rutile TiO_2_ (3.02 eV). This can be attributed to the quantum size effect of the small nanorods. The other peaks at approximately 400 nm (approximately 3.1 eV) are due to the larger size of flower blossom-like structure particles. The adsorption of incident light at nearly 400 and 535 nm in the UV–vis spectra is due to the light absorption by the CdS/CdSe/ZnS QDs.

**Figure 7:**
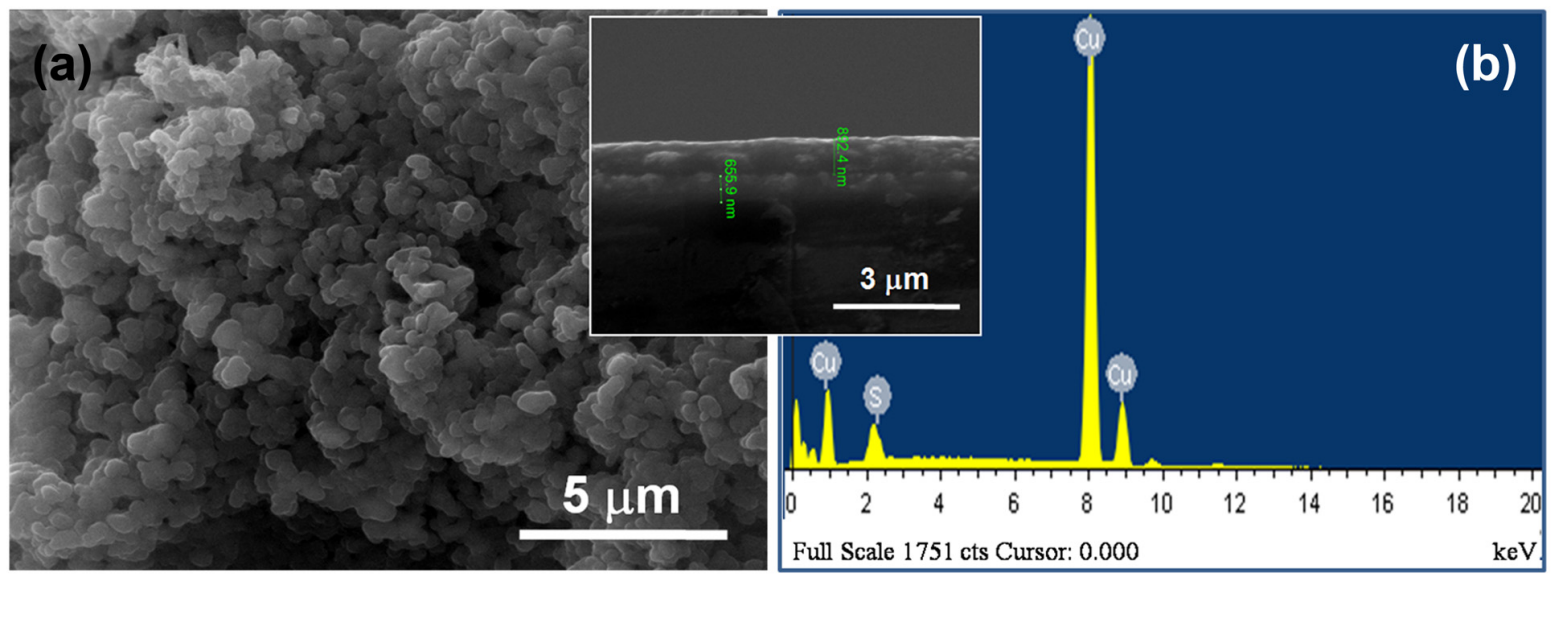
**SEM images and EDS analysis of CuS films. (a)** SEM images of CuS films on FTO, top view, and **(b)** EDS analysis of CuS film deposited on FTO glass.

Figure 7a shows the SEM images of CuS film which is prepared by chemical bath deposition method on the FTO glass. Herein the film is composed of aggregated particles about 1.0 μm in diameter (top view) to form a porous film (side view) (about 892.40 nm in thickness) (inset). To investigate the copper sulfide growth on the substrate, the spectrum of Cu and S elements in EDS analysis is present (Figure 7b) and can be proved that the formation of CuS layer on FTO glass surface can be achieved.

**Figure 8:**
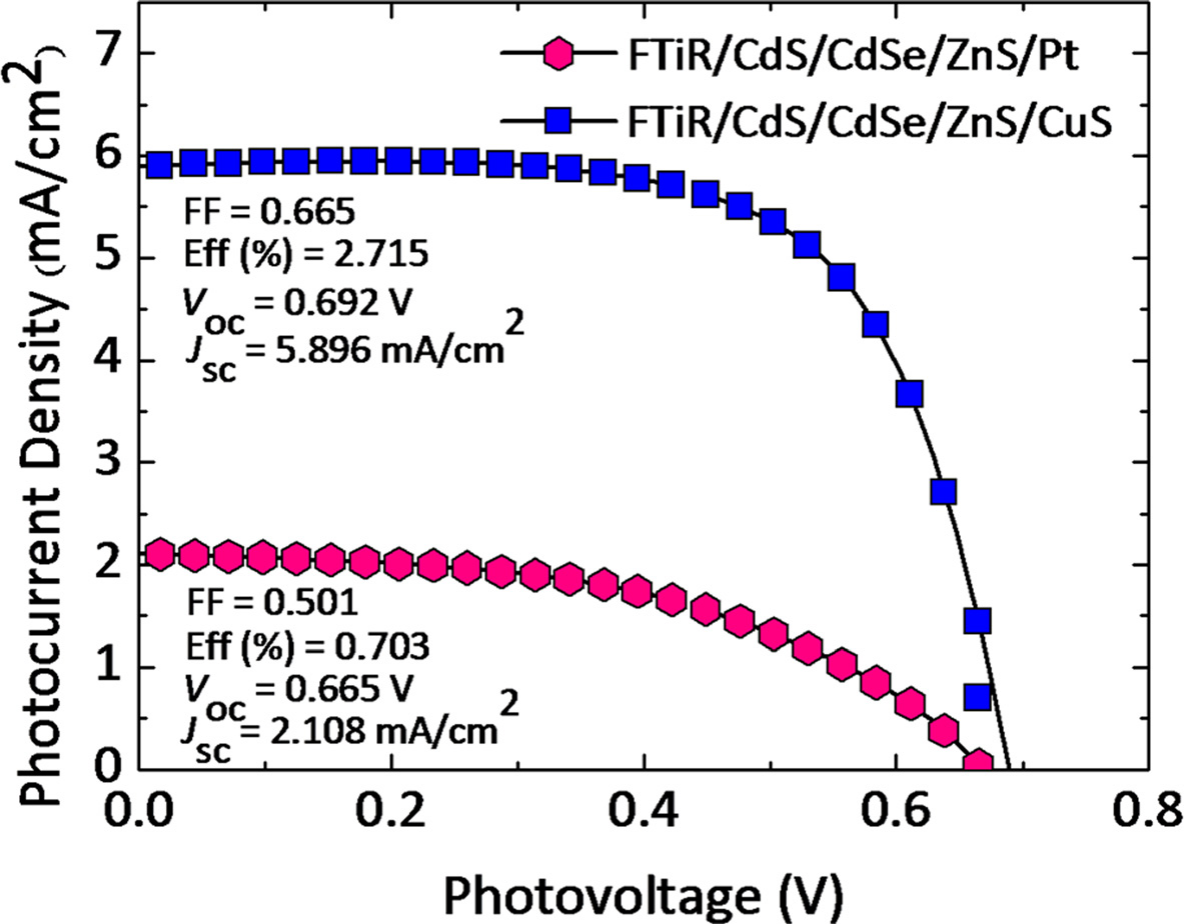
**The photocurrent-voltage (** ***J-V*** **) curves of the assembled QDSSCs.** The photocurrent-voltage (*J-V*) curves of the assembled QDSSCs having the FTiR photoelectrodes performance on the CuS and Pt counter electrode.

Figure 8 shows the photocurrent-voltage (*J-V*) curves of the assembled QDSSCs having the Pt and CuS photoelectrodes when measured under an illumination of 1 sun (AM 1.5, 100 mW cm^−2^). The performance parameters of the solar cells, including open circuit potential (*V*oc), short circuit current (*J*sc), fill factor (FF), and power conversion efficiency (*η*) can be seen in Figure 8. It is evident that the performance parameters of the solar cells have been greatly improved by using the CuS counter as the electrode when compared to that with the Pt electrode.

### Photovoltaic performances of the QDs/FTiR photoelectrodes on the CuS and Pt counter electrode

It should be noted that the CdS/CdSe/ZnS QD-loaded FTiR photoanodes with a CuS counter electrode exhibit a *J*sc of 5.896 mA/cm^2^, *V*oc of 0.692 V, and a fill factor (FF) of 0.665, giving a power efficiency (*η*) of 2.715%. In comparison, the QD-sensitized FTiR cells having Pt as its counter electrode had a *J*sc = 2.108 mA/cm^2^, *V*oc = 0.665 V, a fill factor (FF) = 0.501, and *η* = 0.703%. This shows that the CuS counter electrode for CdS/CdSe/ZnS QD-sensitized photoelectrode made with the FTiR substrate possessed higher efficiency of about 3.72-fold larger when compared to a cell having Pt as its counter electrode. Considering the overall performance parameters, the QD-sensitized FTiR sandwich having CuS as its counter electrode is better than the QDSSCs having Pt as the electrode. This may be due to the greater catalytic activity between the CdS/CdSe/ZnS QD-sensitized FTiR photoanode and the polysulfide electrolyte when CuS is used as the counter electrode. The faster S_2_^2−^ reduction rate on the CuS electrode can accelerate the regeneration rate of QDs. The use of the Pt electrode would lead to a more inferior catalytic activity with the lowest current density and open circuit voltage for the S_2_^2−^ reduction since the surface activity leading to interaction with the polysulfide redox coupled is poor. To explain the phenomena, Lee et al. [30] pointed out that the polysulfide (S_*x*_^2−^) also plays as an electron acceptor to receive electron from the counter electrode through the following reaction: S*x*^2−^ + 2e → S*x*_− 1_^2−^ + S^2−^ which describes the adsorption of sulfide on the surface of a QDs. This reaction plays an important role in the hole-recovery and electron–hole separation. Both effects are expected to give a better hole-recovery rate and, therefore, lead to a higher efficiency of the cell. In support of this hypothesis, we note that the performance of solar cells based on the CdS/CdSe/ZnS QD-sensitized FTiR photoanode and polysulfide electrolyte is greatly improved when using the CuS counter electrode and then when using the Pt counter electrode. However, using the CuS counter electrode on the performance of CdS/CdSe/ZnS QD-sensitized FTiR photoanode, we obtained only 2.715% of power conversion efficiency which is rather low compared to result from other groups (*η* of 3% to 6%) [24-26]. We believe that there is hope for more improvement in the efficiency through the optimization of the FTiR/CdS/CdSe/ZnS photoanode by improving the QD adsorption time and changing the surface morphology of the individual 3D nanostructured TiO_2_ nanorods. The latter will increase the surface area allowing for more QD loading and for an ordered pathway for faster electron transport. This will lead to an enhancement of the light harvesting which will improve the solar-to-electric conversion. In addition, the catalytic activity for redox couple may also be improved by controlling the thickness, morphology, and conductivity of CuS counter electrode and is also an important area of our investigation. The influences of these three parameters for increasing the electrocatalytic activity have been studied [24-26], and noticeable improvement in the power conversion efficiency (up to *η* of 4% to 6%) has been seen.

**Figure 9:**
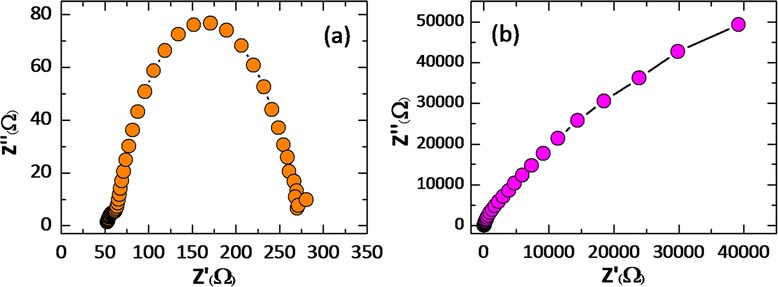
**Impedance spectra of the assembled QDSSCs.** Impedance spectra of the assembled QDSSCs having the FTiR photoelectrode performance on the **(a)** CuS and **(b)** Pt counter electrode.

To see if the above activity is actually occurring, we have measured the electrical contact between the photoelectrode and the electrolytes in the QDSSCs using electro-chemical impedance spectroscopy (EIS). We find that the impedance spectra of the QD-loaded FTiR matched to either a CuS counter electrode or to Pt counter electrode under forward bias (−0.7 V) and dark conditions are quite different. The Nyquist plot (plot of the imaginary part of the impedance vs. the real part) of the EIS of QD/FTiR/CuS (Figure 9a) solar cell appears to be two semicircles (a small one at low frequency and a dominant one at higher frequencies). The values of the series resistance (the *Rs* at intercept of the frequency with the real axis) for the QD/FTiR/CuS (52.10 Ω) will be higher than those of those of junctions having Pt (29.94 Ω) as the counter electrode.

The reason for this is the poorer conductivity of CuS when compared to that of Pt. The large semicircle corresponds to electron injection at the CuS/electrolyte interface and to the transport in the electrolyte at high frequencies (*R*1), meaning that transfer time is due to two processes, transport to from the CuS/electrolyte interface and the electron transfer at the TiO_2_/QDs interface, i.e., the TiO_2_ film (*R*2) [28]. The EIS results for the QD/FTiR/Pt solar cells under the same conditions (Figure 8b) exhibit the signature of the capacitive nature of the system. The capacitive nature is due to the buildup chemical potential which is caused by charge accumulation in the surface traps [31]. The results reported here for the EIS results of QD/FTiR/CuS solar cells are similar to the results reported by Yang et al. [21-26]. They proposed that the relative contribution to the impedance associated with the electron transfer at the counter electrode/electrolyte interface (*R*_1_) could be determined from the radius of the semicircle. From this, they concluded that there were increases in the electrocatalytic activity at the CuS or CoS counter electrode [21-26], compared with that at a Pt counter electrode. The use of these two materials as the counter electrode leads to an acceleration of the electron transfer process at the interface. The increase in the power conversion efficiency is due to the increased electron transfer. We believe that the arcs arise from the electron transfers which occur at the electrolyte-counter electrode interface. The values of charge transfers R of Pt and CuS counter electrodes were 3.97 × 10^3^ and 206.30 Ω, respectively. As a result, the lower charge transfer resistance of CuS CE interpreted as a better electro catalytic activities.

## Conclusions

In present study, the FTiR structures have been synthesized through a double hydrothermal process. This process will lead the FTiR structure to have a higher specific surface area (81.87 m^2^/g). After using the CdS/CdSe/ZnS QDs to sensitize the FTiR photoelectrodes, it was seen that the light absorption shifted to higher wavelengths and that there was a noticeable improvement (about a 3.72-fold improvement) in the power conversion efficiency when CuS instead of Pt was used. The actual improvements were an increase from *η* = 0.703% for the QDSSCs with the FTiR/Pt photoanodes to *η* = 2.715% QDSSCs with the FTiR/CuS photoanode. To explain this, we proposed that the CdS/CdSe/ZnS QD-sensitized FTiR coupled to a CuS counter electrode has the higher electrocatalytic activity while the QDSSC coupled to Pt has a capacitor-like behavior.
